# A Critical Review of Snail Shell Material Modification for Applications in Wastewater Treatment

**DOI:** 10.3390/ma16031095

**Published:** 2023-01-27

**Authors:** Nguyen Thi Hong Nhung, Vo Dinh Long, Toyohisa Fujita

**Affiliations:** 1 School of Resources, Environment and Materials, Guangxi University, Nanning 530004, China; 2 Institute of Environmental Science, Engineering and Management, Industrial University of Ho Chi Minh City, Ho Chi Minh City 700000, Vietnam; 3 School of Chemistry and Chemical Engineering, Guangxi University, Nanning 530004, China

**Keywords:** sea material, snail adsorption, modified biochar, factors affecting, water contamination

## Abstract

Sea material is becoming increasingly popular and widely used as an adsorbent in wastewater treatment. Snail shell, a low-cost and natural animal waste material, has been shown to have a high calcium content (>99%) and a large potential surface area for the development of sustainable adsorbents. This paper presents a novel synthesis of methods for using snail shell absorbent materials in the treatment of wastewater containing heavy metals, textile dyes, and other organic substances. Modified biochar made from snail shells has gained popularity in recent years due to its numerous benefits. This paper discusses and analyzes modification methods, including impregnating with supplements, combining other adsorbents, synthesis of hydroxyapatite, co-precipitation, and the sol–gel method. The analysis of factors influencing adsorption efficiency revealed that pH, contact time, temperature, initial concentration, and adsorbent dose all have a significant impact on the adsorption process. Future research directions are also discussed in this paper as a result of presenting challenges for current snail adsorbents.

## 1. Introduction

Water plays an extremely important role for humans as well as all other creatures on the planet [1]. However, the water environment is becoming increasingly depleted [2,3] and polluted as a result of economic development, such as industrial parks, factories, and agriculture [4,5]. Water pollution contains a lot of pollutants that affect the environment and human health [6]. Textile dyeing wastewater is one type of wastewater that has a significant impact on human health [7] and the aquatic environment [8]. The discharge of colored waste in natural water bodies has been prohibited under new environmental regulations governing textile products. Therefore, the cost-effective treatment of effluents containing a variety of textile dyes has become a requirement for clean production technology in the textile industry [9].

Furthermore, heavy metals are the most commonly found contaminants in wastewater that are a public-health concern due to their high toxicity [10]. Sources of heavy-metal pollution include mining waste [11,12], water leaks in landfills [13], municipal wastewater, and industrial wastewater [14], especially from industries, such as electroplating and electronic metal fabrication [15]. Heavy metals are necessary for human metabolism; however, high concentrations can be toxic and dangerous to humans [16,17]. Even more dangerously, if the body accumulates large amounts of heavy metals, it will cause many serious complications, including brain damage, shrinking muscle bundles, deformed fingers, legs, and joints, patients going insane, and death after a few hours to several months or years of exposure [18,19]. Therefore, studying the presence of heavy metals and how to treat them in polluted areas has been of great interest in developing countries [20,21]. Technologies for wastewater treatment of metals and textiles include biological [22] membrane [23] adsorption [24,25], using phosphonium-based ionic liquids [26]. However, these processing technologies are relatively expensive and complex in the treatment process.

**Figure 1:**
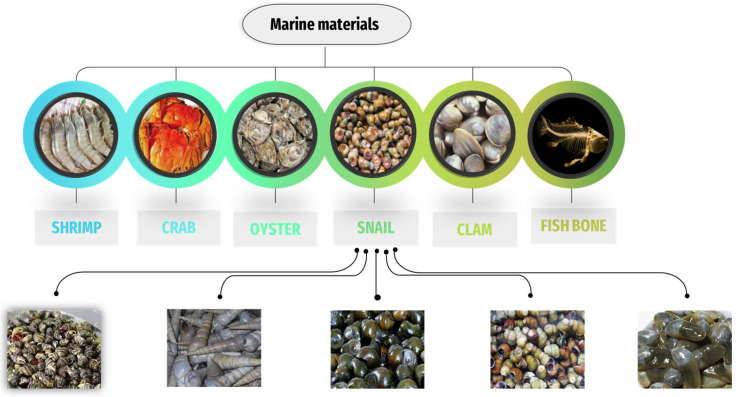
Popular marine materials in recent years in the application of adsorbents for wastewater treatment.

Marine animal farms are becoming more common in countries, particularly in Asian countries, such as Vietnam, China, and Thailand [27,28]. Food waste from marine animals is increasing day by day because food from the sea is always popular with people around the world [29]. Waste is treated from materials that have an abundant and low-cost source of calcium to be considered a good source of materials for waste treatment in the environment [30]. Due to their high calcium oxide content, various seashells have been studied and found to be similar to limestone [31]. Therefore, many studies have been conducted to investigate how to use these waste species to create materials for waste treatment in the soil and water environment, thereby lowering waste-treatment costs while also benefiting environmental treatment. The most recently studied marine materials in terms of using their wastes as adsorbents in wastewater treatment are shown in Figure 1.

There are many studies using adsorbent materials to treat different types of waste, such as heavy metals [32,33], antibiotics [34], inorganic, and organic compounds [35], in which the use of various types of adsorbents, such as agricultural wastes [36,37], industrial waste [38,39], environmental pollutants, such as fly ash [40,41], peat [42,43], blast furnace slag [44,45], and graphite materials [46], has been considered. However, in recent years, the use of adsorbent materials from the sea has been focused on due to treatment efficiency as well as solving environmental problems [47]. Shrimp [48,49,50], crabs [51,52], oysters [53,54], snails [55,56], clams [57,58], fish bone [59,60], and other marine species [61,62,63] are used as highly effective adsorbents in the removal of wastes from water pollution.

**Figure 2:**
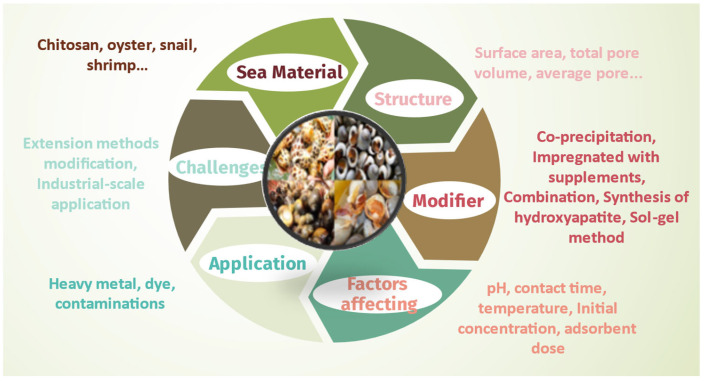
General overview of snail shell materials for wastewater treatment.

The use of snail shells is one of the effective and new adsorbent materials in textile dyeing and heavy-metal wastewater treatment, according to a survey of many recent research documents. There are many studies on using activated carbon from snail shells to absorb pollution in wastewater [55,64], but there are not many general reports on biochar modification from this material, especially for wastewater treatment of heavy metal and textile dyeing. Figure 2 summarizes the snail shell materials analyzed in this research for wastewater treatment.

A summary of review articles published in the last few years, as shown in Table 1, focuses on chitin/chitosan materials, mainly chitosan synthesized from chitin/chitosan, shrimp or oyster shells, for which there is no specific overview and focus on snail shell materials. Furthermore, there is a diversity of different types of snails for shell material, as well as specific characteristics of each species that will affect the adsorption of pollutants in wastewater. Therefore, a comprehensive article on separate snail shell materials in waste adsorption, focusing on heavy metals, textile dyeing wastewater, and other wastewater, is required to provide an overview of this potential material.

Furthermore, this paper analyzes the mechanism and process, as well as different methods, in biochar modification, comparing and discussing the effectiveness of the methods and, thus, providing an overview of biochar modification on snail shells in the use and modification of snail shell materials for wastewater treatment. This article is analyzed and discussed using a process of synthesizing and researching from over a hundred different articles and statistics in the form of tables to provide an overview, which is easy to understand for future readers.

## 2. Property, Structure, and Process of Snail Shell as Bio-Adsorbent

Many different types of snails have been studied as adsorbents in freshwater [82] and saltwater [47] in many areas of the world. Shells help mollusks survive in extreme conditions, protecting them from the tremendous pressure on the seabed [83]. However, humans use snails only for meat through the food chain, and such a large amount of shells creates a large amount of waste for the environment [84]. The characteristics of snail shells were analyzed using FTIR, SEM, MAP, EDAX, and BET analyses in most studies [85]. The findings of the shell material composition analysis almost entirely include the ingredients listed in Table 2, including ash, calcium, fiber content, fat... and some heavy metals or minerals [86]. The calcium carbonate content of snail shells is extremely high, possibly exceeding 95% [87]. The amount of calcium in the shells of each species varies depending on the thickness of the shell as well as morphology and other environmental factors [88]. The calcium carbonate concentrations in five different species of snails collected in different lakes in the United States range from 97 to 98.8% [88]. Considering the structure of a freshwater snail of *Physa* sp., the results of morphology and crystalline characterization of calcium carbonate polymorphs show calcium, as the main element, as well as sulfur, phosphorus, and other elements [89].

The ash content shows the existence of carbon compounds and inorganic components in snail shells, which plays an important role in becoming adsorbents in water treatment [90]. Analysis of heavy-metal composition in snail shells found that iron content was highest for other metal components, such as zinc, manganese, copper, and iron [91]. Other minor components, Cr, P, Al, Ni, Mg, Si, and K, also exist in snail shells [92,93]. This is also a demonstration of the effective use of snail shells as coagulant materials in wastewater treatment. According to Saida Parveen’s research on physical and chemical analysis of three different freshwater snail species, the shells of these snails can make strong and mechanically sustainable biological materials applied in various fields including bioremediation [94]. The structure of the snail shell varies in shape, size, and shell layers to different species depending on the geographical location and characteristics of the snail [89,95].

**Table 2:** Snail shell material structure for absorbing different types of waste.

Snail Name/Country	Modifier/Method	Porosity (°C)	Structure	Components (%)	Adsorption	Ref.
Snail (Nigeria)	Furnace to powder	200, 300 and 400	Surface area: 0.99 m^2^/g	-	Pb(II)	[53]
*Achatina achatina* (African)	Co-precipitation	-	Moisture content: 2.1%	Ash: 93.76, Calcium: 99.74	Aniline blue	[96]
*Achatina achatina* (African)	No	-	-	Protein: 0.12, Fiber: 4.06, Fat: 0.79, Ash: 2, NFE: 93.04	Wastewater from the brewery industry	[91]
*Archatina maginata* (African)	No	-	-	Protein: 0.42, Fiber: 3.37, Fat: 0.75, Ash: 10, NFE: 82.36	Wastewater from the brewery industry	[91]
*Archatina fulica* (African)	No	-	-	Protein: 0.3, Fiber: 3.96, Fat: 0.38, Ash: 10, NFE: 82.36	Wastewater from the brewery industry	[91]
*Limucalaria* sp. (African)	No	-	-	Protein: 0.23, Fiber: 4.14, Fat: 0.48, Ash: 13, NFE: 82.15	Wastewater from the brewery industry	[91]
*Pomacea canaliculata* L. (Indonesia)	No	900	-	O: 63.28, P: 11.79, Ca: 24.93	Pb (II)	[97]
*Pomacea canaliculata* L. (Indonesia)	Hydroxyapatite-SiO_2_ composite	900	-	O: 58.17, P: 10.33, Ca: 22.16, Si: 9.34	Pb (II)	[97]
*Bellamya chinensis* (Vietnam)	No	-	Surface area <2 m^2^/g, total pore volume <0.001 cm^3^/g	C: 28.15, O: 62.68, Ca: 9.57,	Cr (VI)	[98]
*Bellamya chinensis* (Vietnam)	Impregnating with iron oxide	-	Surface area: 69.69 m^2^/g, total pore volume: 0.104 cm^3^/g	C: 6.05, O: 70.84, Cl: 9.31, Ca: 6.64, Fe: 7.16	Cr (VI)	[99]
*Rostellaria* (Iraq)	No	-	Surface area: 295 m^2^/g, moisture content: 24.33%	CaO: 52.7, SiO_2_: 2.4, Al_2_O_3_: 0.68, Fe_2_O_3_ 0.44, MgO 1.5, SO_3_ 0.28	Azure A, B Dye	[100] [102]
*Umbonium vestiarium* (Iran)	No	-	Surface area: 17.01 m^2^/g, pore volume: 0.038 cm^3^/g, pore size: 90.42 Å	-	Co (II)	[102]
*Helix pomatia* (Nigeria)	ZnCl_2_	500–800	Moisture content: 1.75%	Ash: 85	Methylene blue	[103]
*Helix pomatia* (Nigeria)	CaCl_2_	500–800	Moisture content: 1.75%	Ash: 91	Methylene blue	[103]
Snail (Nigeria)	No	500	Surface area: 2567.32 m^2^/g, moisture content: 0.32%, porosity: 48%	Ash: 12.5	Wastewater from beverage	[104]
Snail (Nigeria)	Activating agent of H_3_PO_4_	500	Surface area: 2987.69 m^2^/g, moisture content: 0.27%, porosity: 72%	Ash: 7.3	Wastewater from beverage	[104]
Snail (Nigeria)	Coagulant aid in the alum precipitation	-	Bulk density: 1.33 g/cm^3^, moisture content: 2.1%,	Ash: 93.76 Ca: 99.74, Mg: 0.0002, Na: 0.0008 K: 0.0009, Cu: 0.00002, Pb: 0.0005	Malachite green	[105]
Snail (China)	Mixture with activated chestnut shell	500, 700, 900	Surface area: 1705 m^2^/g average pore diameter: 4.07 nm	C: 35.63, O: 42.31, Ca: 22.05	Methylene blue	[106]
*Physa acuta* (India)	No	550	Water content: 886.1 mg/g	Ash: 54.95, CaCO_3_: 98.9	Cd(II)	[107]
*Argopecten irradians* (China)	No	-	Surface area: 1.88 m^2^/g, pore size: 10.49 nm, pore volume: 0.005 cm^3^/g	-	Cr(VI) and Cu(II)	[108]
*Argopecten irradians* (China)	Calcinated	300	Surface area: 2.78 m^2^/g pore size: 14.86 nm pore volume: 0.01 cm^3^/g	-	Cr(VI) and Cu(II)	[108]
*Argopecten irradians* (China)	Acidification shell	300	Surface area: 2.75 m^2^/g pore size: 16.19 nm pore volume: 0.01 cm^3^/g	-	Cr(VI) and Cu(II)	[108]
Snail (Morocco)	No	700, 900, and 1200	Surface area: 0.72 m^2^/g	-	Cu (II)	[109]
*Anadara uropigimelana* (Egypt)	No	-	Surface area: 2.82 m^2^/g	C: 11.1, O: 41.1, Ca: 47.6	Methylene blue	[110]

Snail shells are typically modified after being pre-treated through washing and drying processes to increase surface and adsorption area. Some unmodified pretreated materials have an adsorption surface of less than 2 m^2^/g and 0.001 cm^3^/g with BET and total pore volume [99]. The average surface area of the unmodified shell material is significantly lower than that of the modified biochar, as shown in Table 2. A study on the modification of snail shell materials by impregnation with Fe found that the shell material increased to over 69.69 m^2^/g and 0.104 cm^3^/g for the surface area and total pore volume, respectively [111]. To evaluate the degree of adsorption structure of snail shells, three types of shell powder were studied: raw material, calcined material, and acidic-treated calcined material. The results revealed that the structure of the calcined materials had a larger specific surface area than the raw materials, and the acidic-treated calcined materials had a larger pore size and volume than the calcined materials [108]. Therefore, recent research mainly focused on modification improvements to create optimal materials that give the highest performance in wastewater treatment. Based on surface area properties, the adsorption capacity of snail shells demonstrates that shells have a high ability to treat wastes, such as heavy metals, textiles, and other wastes [106]. According to Stevens et al., the adsorption efficiency of snail shells for Pb^2+^ is higher than that of oysters and periwinkles based on material properties [53]. A study on the synthesis of sea materials showed that the shell material also has very good characterization results compared to other chitosan materials made from materials, such as crab, lobster, and squid [112].

The biochar production process consists of three stages: pre-pyrolysis, main pyrolysis, and after pyrolysis [112]. After the shell is recovered, it will undergo treatment and pyrolysis to form a material that can adsorb waste. Most snail shells need to go through this important pyrolysis step to become an adsorbent. After being washed and dried under various conditions and temperatures ranging from 200 to 1000 °C, all shells will be soaked in an acid solution to remove unnecessary substances. The snail shell processing will affect the efficiency of the adsorption process. Table 3 is a summary table of different shell-processing processes to form materials for the adsorption process.

Pyrolysis can be used to process shells at a variety of temperatures. In some other treatments, however, the snail shell is finely ground before being dried and screened. Most studies will select a sieve size that is not too large because too-large particles will not be able to absorb much of the pollutant. A study on particle-size classification in Cadmium wastewater treatment found that particles smaller than 200 μm perform better than particles between 200 and 500 μm [107].

**Table 3:** Snail shell material processing methods.

Name	Dry Condition (°C/Time)	Temperature (°C)	Time Furnace (h)	Reagent	pH	Adsorption	Ref
Snail shell-rice husk	100/24 h	681.1	2.61	-	9	Brilliant green dye	[113]
*Pomacea canaliculata* L.	In the sun/24 h	900	4	(NH_4_)_2_HPO_4_		Pb(II)	[97]
*Solamen Vaillanti*	100/0.5 h	400	3	H_3_PO_4_ solution	6.5	Cu(II), Co(II), and Pb(II)	[114]
Fresh snail shell	80/24 h	-	-	H_2_SO_4_ solution	2	Cr (VI)	[98]
*Rostellaria*	100/24 h	-	-	-	6.8	Azure B dye	[100]
*Umbonium vestiarium*	105/24 h	1000	4	-	7	Co (II)	[102]
*Helix pomatia*	105/3 h	500–800	3	ZnCl_2_ and CaCl_2_	-	-	[103]
Snail	110/2 h	500	1	H_3_PO_4_ solution	7.04		[104]
*Oncomelania hupensis Gredler*	80/24 h	500, 700, and 900	1	H_3_PO_4_ solution	4.7	Methylene Blue	[106]
*Physa acuta*	50/1 h	-	-	-	-	Cd(II)	[107]
*Argopecten irradians*	60/48 h	200, 300, 400, and 500	3	HCl solution	-	Cr(VI) and Cu(II)	[108]
Snail shell	100/12 h	1000	1	-	-	Cu(II)	[109]

Various model types are analyzed and evaluated to determine their suitability for the waste treatment of snail shells. The most-adapted models include Temkin, Freundlich, and Langmuir adsorption isotherms [53].

According to the survey results on kinetic model analysis, the three most-studied types are pseudo-first order, pseudo-second order, and intraparticle diffusion, with pseudo-first-order kinetics being the best fit with the results of other remaining types. The adsorption kinetics of magnetic shell materials impregnated by LP. Hoang were well described by the pseudo-second-order model and the best-fitting model that described the Cr(VI) adsorption isotherm was the Langmuir model [111]. Similarly, studies on the modification of shell material via the sol–gel method also showed Langmuir isotherm and pseudo-second-order kinetics that are consistent with the studies.

However, in some cases, depending on the pollution characteristics, there are different suitable models for research. In the same study, using snail shells to treat Cr(VI) and Cu(II) ions from wastewater, the Freundlich model was appropriate for Cr(VI) adsorption, whereas the Langmuir model was appropriate for Cu(II) adsorption [108].

## 3. Popular Biochar-Modification Methodologies for Snail Shell Materials

There are many different biochar-modification methods. Common methods are listed in Table 4.

- Impregnated with supplements: For this method, the snail shells are washed to remove unnecessary substances and dried. Then, they will be soaked with a solution to increase the adsorption process. After being soaked in biochar, substances will experience interactions between the admixture molecules and calcium carbonate, resulting in the release of carbon molecules and a change in the structure of the adsorbent [99]. Many studies have used different types of substances and varied mixing ratios, such as phosphoric acid [114], FeCl_3_ [99], ZnCl_2,_ and CaCl_2_ [103]. A study using Fe as an impregnated agent in combination with a snail shell revealed that the effect of Fe_3_O_4_ formed on the material was very good and had an expected effect on Cr(VI) adsorption in wastewater [111].
- Combination of other adsorbents: The incorporation of other adsorbent materials into snail shell biochar is a current trend when the specific adsorption efficiency of the shell material is not high. The substances associated with the snail shell material come from a variety of sources, the majority of which are agricultural waste. Snail shell and rice husk were combined and calcined for more than two hours at 681.1 °C to produce mixed adsorbent materials with quite high adsorption results when compared to individual materials [113]. Another study on the combination of snail shell and chestnut using a simple combination method and different ratios of snail shell and chestnut resulted in methylene blue treatment efficiency of up to 92% [106]. A combination of snail shell and pine cone powder also demonstrated high potential for heavy-metal treatment in wastewater due to the combination of cellulose in an amorphous crystalline phase and calcium carbonate compound [115].
- Synthesis of hydroxyapatite: This method involves heating the shell to a high temperature to convert CaCO_3_ to CaO phase; the process can be supplemented with a variety of acidic solutions [97]. The thermal treatment range has a large range of values to find the right temperature for each shell. The results of a temperature range study from 200 to 1000 °C to compare the particle evaluation of different raw materials and pyrolysis materials revealed that a calcined snail shell at 200 °C was aragonite polymorphs, calcite at 400–600 °C, and calcium oxide at 800–1000 °C [92].
- Co-precipitation: Because wastes from sea material have a high calcium carbonate content, using these wastes for research as an adjunct to co-precipitation is considered a method with high efficiency. This method has the advantage of being able to use a wide range of materials, adapt to a wide range of reaction conditions, and produce particles with relatively even, uniform, and small sizes. The shell material can be considered an effective adsorbent as a coagulant aid due to its high iron content for the easy high-coagulating property [116]. N.A. Oladoja’s research on African snails used as a coagulant aid in alum precipitation to treat dye molecules from wastewater produced better results than treating alum and shell precipitates separately. In addition, the sludge obtained from this co-precipitation has better properties than the sludge obtained using the precipitate alone [96]. However, this method of co-precipitation also has some limitations in terms of time consumption; if co-precipitates have different precipitation rates or trace impurities, this can also cause precipitation [117]. Another study showed that using snail shell material and alum alone does not bring good results in the treatment of malachite green; however, using snail shell as a coagulant combined with alum precipitation results in a much higher processing result, increasing efficiency [105].
- Sol–gel method: This method has been widely used in past years because of its advantages and is also used for other types of sea material, as studied by Tetyana M Budnyak et al. [118] or research by Guillermo J. Copello et al. [119] on the synthesis of chitosan–silica materials via the sol–gel method. A study on snail shell in situ hybridization of different dyes via the sol–gel method showed that the treatment efficiency of Congo Red (>95%) was higher than that of Methylene blue (<80%) at an initial waste concentration of 100 mg/l. The synthesis of hydroxyapatite with Silica gives a better Pb(II) adsorption efficiency than that of hydroxyapatite from snail shells of 123.46 and 135.14 mg/g, respectively.

**Table 4:** Waste treatment efficiency of shell materials with different methods.

Type of Snail	Method	Pollutant	Concentration of Pollution (mg/L)	Efficiency (%)	Remarks	Ref.
*Achatina achatina*	Co-precipitation	Aniline blue	100	>95	Evaluation of the effect of pH, time, and sludge settling on textile dyeing waste removal	[96]
*Achatina Achatina*	Sol–gel	Methylene blue and Congo Red	100	>95	Investigate the influencing factors on initial waste concentration. pH is not affected, but sludge settlement is affected by waste removal	[120]
*Bellamya chinensis*	Impregnating of iron oxide	Cr (VI)	60	76.8	The adsorbent material has the characteristics of CaCO_3_ and Fe_3_O_4_ to increase the adsorption surface compared to unmodified material, suitable with Langmuir and Pseudo-second-order model	[111]
*Solamen Vaillanti*	Impregnating	Cu(II), Co(II), and Pb(II)	10	94.4, 96.5, and 96.7	Evaluation of the removal of three different heavy metals present in real wastewater and the influence of factors including pH, temperature, contact time, waste initial concentration, and adsorbent dosage	[114]
Golden Snail Shell	Sol–gel	Pb(II)	25	97.1	Comparison of composites hydroxyapatite with SiO_2_ and hydroxyapatite from shells shows that modification material gives better performance	[97]
*Helix pomatia*	Impregnating of ZnCl_2_ and CaCl_2_	Methylene blue	1000	98 and 67	Evaluated as a raw material for the production of activated carbon with ZnCl_2_ and CaCl_2_ at temperatures ranging from 500 °C to 800 °C	[103]
*Oncomelania hupensis Gredler*	Combination of other adsorbents	Methylene blue	1300	92	The mixture of activated chestnut shell biochar and pyrolyzed snail shell material in a simple process for high-concentration wastewater treatment	[106]
*Rostellaria*	Mixing with melamine	Azure A dye	5	93.9	Compare the adsorption capacity of snail shell- Melamine Complex and this polymer modification biochar based on the addition of formaldehyde.	[121]

The results show that modifying the shell materials results in significantly higher adsorption than untreated ones. The adsorption capacity of Rostellaria snail shell for Azure A dye was 89.5% [122], Azure B dye was 83% [100], and Malachite Green was 86.66% [101], however, if the material is modified up to 93.9% when treating Azure A in wastewater [121].

## 4. Effect of Nutrient to Adsorption Biochar

Many factors influence the efficiency of adsorbent adsorption of pollutants in water, including solution pH, contact time, temperature, initial concentration, adsorbent dose, and nutrients. Recent studies on the effects of snail shell materials on the adsorption of pollutants in the aquatic environment are summarized in Table 5.

### 4.1. pH

pH greatly affects the adsorption process of almost all adsorbents. Under different pH conditions, there will be different adsorption efficiency, influence, and adsorption efficiency of textile dyeing wastes shown in the table below. Investigating the influence of pH on adsorption efficiency within a very large range from 2 to 12, Cr(VI) adsorption studies showed the highest efficiency at pH 2 [98]. Another study on Cd^2+^ adsorption found that the optimal pH was 6 rather than low pH with high protonation [107]. The functional groups are protonated and become positive under acidic pH conditions. Cationic dye adsorption is favored at higher pH levels, whereas anionic dye adsorption is favored at lower pH levels [51]. Similarly, An et al. discovered that as the pH increased, the value of the zeta potential decreased, implying that more functional groups were exposed to the surface material, which facilitated the adsorption process [106].

A coagulant’s chemistry is affected by its pH during coagulation. Hydrolysis products of alum are primarily medium polymers or monomers with a high ability to remove dissolved organic carbon via complex adsorption, charge neutralization, or co-precipitation [96].

### 4.2. Contact Time

Contact time is an important factor influencing adsorption efficiency. The change in contact time will affect both the process equilibrium and the treatment reaction rate. The contact time in the adsorption process was studied from 0 to 240 min in most research. When the contact time increases, the adsorption efficiency increases as well, but after a certain threshold, the effect saturates and no longer increases. When investigating the influence range of 0 to 180 min, Redouane Ouaf et al. found the highest Cu(II) removal efficiency at 90 min in a study on snail shells [109]. A study of Cd^2+^ treatment at pH 6 over a survey period of 10 to 80 min found that treatment efficiency was lowest at 10 min and highest at 60 min. Michelle Castaneda et al. studied the effect of time on the biosorption of Pb^2+^ at pH 5.5 and a temperature of 30 °C, giving the optimal time at 80 min [123]. To fit the kinetic models, different values of waste treatment with time intervals are used.

### 4.3. Temperature

Temperature is the factor that has received the least attention in the survey of factors affecting adsorption efficiency according to Table 5. Almost from the beginning of the pyrolysis of snail shells, studies have chosen a specific temperature to obtain biochar material from snail shells from which to conduct other studies on the degree of influence. However, when the effect of temperature on the structure of the adsorbent is considered, the results show that as the temperature rises, the carbon content also increases [29]. The effect of temperature was studied by Pranesh Paul et al. in four different temperature values ranging from 20 to 35 °C, with the highest adsorption efficiency at 35 °C due to increased calcium content by dissolving material for phosphate processing [124].

### 4.4. Initial Concentration

Studies frequently take the difference between initial concentrations to be very wide, with approximately different values. In contrast to the contact time, the adsorption of Pb ions in wastewater increased as the contact time increased and the initial concentration decreased in solution [53]. As the initial concentration rose, the concentration occupied the adsorbent’s active sites on the surface during the adsorption process, and the active sites on the surface reduced until adsorption saturation was reached. Benliang Zhao et al. discovered that the cadmium treatment efficiency decreased as the Cd temperature gradually increased to 500 mg/L in a study of golden apple snails on cadmium treatment in water [125].

### 4.5. Adsorbent Dose

An important parameter in treatment studies is the amount of adsorbent used. Adsorption of both metals increased as the amount of snail shell materials increased. For textile dyeing wastewater, the textile dyeing wastewater removal efficiency increased when increasing the snail shell dosage per wastewater concentration for studies on low textile dyeing wastewater concentration [96]. According to the findings of Olayinka John Akinyeye et al., in an adsorbent dose range of 0.25 g to 1.5 g for the adsorption of Pb^2+^ and Ni^2+^ ions, different effects will be obtained at different doses. At a 0.25 g dose, it can be 99.81% effective and 99.3% effective at a 0.5 g dose [127]. However, as the adsorbent dose is increased, the adsorption efficiency does not increase, indicating that the maximum effect will occur at a certain dose and cannot be higher. Similarly, Khalid Z. Elwakeel et al. discovered that increasing the adsorbent dosage from 25 to 150 g/L increases methylene blue processing efficiency to a maximum of 93.6% [110].

**Table 5:** Parameters and conditions affecting the heavy-metal adsorption process of snail shell.

Snail Name	pH	Contact Time (Min)	Temperature (°C)	Initial Adsorbent Concentration	Pollutant Concentration (mg/L)	Efficiency (%)	Pollutant	Ref.
Snail	-	10, 20, 30 and 40	200, 300 and 400	100 g/100 mL	30–120	99	Pb ion	[53]
Snail	3–6	10–90	-	0.1–0.7 g/100 mL	5, 10, 20, 50, 100	89.61	Aniline blue	[96]
*Bellamya chinensis*	2–9	20–180	20–40	0.2–3 g/100 mL	30–200	42	Cr (VI)	[98]
*Bellamya chinensis*	2–12	5–240	-	0.04–0.48 mg/100 mL	5–80	76.8	Cr (VI)	[111]
Golden snail	2–10	0–90	-	0.08 mg/100 mL	25–200	97.1	Pb(II)	[97]
*Rostellaria*	2–12	10–140	25–65	0.01–0.08 g	25	83	Azure B Dye	[100]
*Rostellaria*	2–12	10–120	25–65	0.01–0.08 g	5	89.5	Azure A Dye	[122]
*Rostellaria*	2–12	10–120	25–65	0.005–0.08 g	7	86.66	Malachite Green	[101]
*Umbonium vestiarium*	3–9	5–120	-	0.025–0.6 g/100 mL	10–50	93.87	Co (II)	[102]
*Oncomelania hupensis Gredler*	4–12	15, 30, 60, 120, 180, 300, 600 and 1440	500, 700 and 900	20 mg	500, 900, 1300, 1700, 1900 and 2100	92	Methylene blue	[106]
*Physa acuta Asif*	2–7	10, 20, 40, 60 and 80	-	0.2–1 g/100 ml	25–1000	87	Cd(II)	[107]
*Argopecten irradians*	1.5–10	30–390	200–500	1–14 g/100 mL	50–400	32.86	Cr(VI)	[108]
*Argopecten irradians*	-	30–180	200–500	-	100–2000	99.04	Cu(II)	[108]
Snail	2–8	0–180	700, 900 and 1000	0.05–1.2 mg	50–500	99	Cu (II)	[109]
Snail	2–12	10–120	25–65	0.005–0.08 g	30	99.09	Remazol Brilliant Blue dye	[127]
*Hexaplex kuesterianus*	2–9	60–180	-	0.2–1 g	40	94.4	Pb (II)	[128]
*Hexaplex kuesterianus*	2–9	60–180	-	0.2–1 g	10	75.3	Cu (II)	[128]
Golden snail	1.5–5.5	2–100	10–50	0.005–1 g/100 mL	5–500	99.2	Cd(II)	[125]
Snail	1–9	5–360	32–82	0.25–1.5 g	100–500	99.93 and 70.58	Pb (II) and Ni(II)	[126]
Snail	3–11	5–240	20–50	0.04–0.6 g/100 mL	25–55	95	Basic Yellow 28	[129]

In addition to the important influencing parameters listed above, some studies also evaluated the effect of different particle sizes and the ratio of admixture on adsorption efficiency or ionic strength on adsorption capacity. Specifically, in the study of Redouane Ouaf and colleagues, the different particle sizes were evaluated from 50 to 800 μm and the results showed that the effect of particle size on waste removal efficiency was not significant [109]. However, when comparing the effect, it is a good choice for the shell adsorbent for particle sizes larger than 300 μm. The higher the adsorption surface, the smaller the adsorbent particles, specifically at a particle size of 212 μm of the snail shell of *Hexaplex kuesterianus,* resulting in higher adsorption than 400 μm for both heavy metals of Pb (II) and Ni (II) adsorption [128]. The mixing ratio also greatly determines the adsorption efficiency, with adsorbents usually being mixed with different substances under many kinds of ratios. Research on mixing chestnut and snail shells at a ratio of 3:1 gave better results than ratios of 1:3 and 1:1, with methylene blue adsorption efficiency of up to 92% [106]. When different concentrations of NaCl, KCl, MgCl_2_, and CaCl_2_ salts were used to evaluate the effect of ionic strength on the adsorption of Azure A, the influence of CaCl_2_ showed higher results than the remaining salts, but this effect was not significant on the adsorption results [122].

## 5. Challenges and Future Research Directions

From the research results of snail shell materials, it is shown that modification will be a good research direction to increase the adsorption capacity by increasing the surface of the shell material. However, the adsorption results have not yet reached the maximum and are still limited in some studies. Therefore, the combination of snail shell adsorbent and other adsorbents from chitosan or agricultural materials can be considered for further studies.

The research results are also applied to samples tested in the laboratory, where possible, using actual or more-advanced samples applied in real conditions of some production facilities, factories, or industrial zones because real wastewater samples often have many different pollutants and the treatment depends on the reciprocal reaction in the adsorption process. From there, a larger commercial scale can be applied if the actual experimental results are as effective as those found in the laboratory. Furthermore, nanocomposites containing calcium carbonate nanoparticles should be investigated using snail shell materials with a high calcium content.

In summary, based on the results of adsorption, recyclability, economic efficiency, and environmental friendliness, modification methods to improve snail shell adsorbent and adsorption conditions should be considered selectively.

## 6. Conclusions and Remarks

Adsorbents from marine materials, including shrimp, crabs, snails, and fish bones, are currently being recovered and used as cheap and highly effective adsorbents. In particular, the snail shell material is being focused on because of its adsorption capacity for different types of wastewater and its high efficiency. In comparison to other materials from the sea as well as agricultural materials, snail material has a high surface area, indicating the potential for waste adsorption. Although the calcium content of the snail shell is very high, the crystallinity of the shell is higher after being sintered, making the shell easy to precipitate and difficult to dissolve, which is also a disadvantage in handling. Therefore, to increase the adsorption efficiency, most recent studies have used modifications to increase the adsorption surface of the material. The synthesis of hydroxyapatite from snail shell material yields diverse results in terms of morphology and composition from the low to high temperature range. The results show that the average particle size of synthesized hydroxyapatite increases with reaction temperature response. When combining agricultural materials, the material combination shell material promoted the adsorption of textile dyeing wastewater, demonstrating material complementarity. Furthermore, the material combination shell material provides high efficiency through photocatalysis. The impregnation method used before or after pyrolysis altered the structure and functional properties of the adsorbent. In addition, acidic modification is used during shell processing to adjust pH and remove mineral elements. The co-precipitation method has many advantages for preparing adsorbents from snail shells, but it is time-consuming and sometimes has reproducibility issues. In contrast, the sol–gel method has the advantages of composition control, a stable surface, and good adsorption. In addition, the factors of pH, temperature, catalyst, initial pollutant concentration, and adsorption time also affect the adsorption process of pollutants in the aqueous environment.

## Figures and Tables

**Table 1:** Summary of review articles on wastewater treatment related to sea material in recent years.

No.	Type of Material	Year	General Content	Pollution Adsorption	Ref.
1	Chitosan-based adsorbents	2019	Summarize heavy metal ion desorption and potential regeneration of chitosan-based adsorbents using different desorption agents	Heavy metal	[65]
2	Chitosan Modifications	2020	The trend in chitosan modification and adsorption capacity of cross-linked chitosan-based materials	Metal ion, dye, and pharmaceuticals	[66]
3	Sea material shells: oyster, snail, and shrimp shell	2020	A brief description and literature review of the heavy metal ion adsorption process’s equilibrium, kinetic, and thermodynamic behaviors	Heavy metals	[47]
4	Marine-shell	2020	Studying the mechanisms of heavy metal absorption with different pyrolysis conditions of biochar materials	Heavy metals	[67]
5	Chitosan	2020	Different chitosan modifications and their applications in water and soil pollutant removal	Heavy metals, dyes, antibiotics, pesticides, and biological pollutants	[68]
6	Chitosan/chitin-carbonaceous material composites	2020	A review of the preparation of chitosan/chitin-carbonaceous material composites, adsorbent regeneration, and reusability	Heavy metals, dyes, and other contaminants	[69]
7	Chitin and chitosan-based biomaterials	2021	Detailed analysis of chitin and chitosan adsorption property modifications	Textile dyes	[70]
8	Chitosan-based adsorbents	2021	Detail on chitosan modification methods and the influence of co-existing ions on the synthesis processes, adsorbent efficiency, and regeneration methods	Heavy metal	[71]
9	Chitosan and chitosan bio-adsorbents	2021	The effects of chitosan and its derivatives on preparation strategies, adsorbent structure modification, and adsorbent variables using batch and fixed column studies	Nitrogen-containing pollutants	[72]
10	Hydrogels based on chitosan and alginate	2021	The use of chitosan and alginate in biobased hydrogel adsorbents and potential combinations with other ingredients	Dyes and metal ions	[73]
11	chitosan and its derivatives	2021	Preparation of chitosan and its derivatives and their application in wastewater treatment	Heavy metals	[74]
12	chitosan-based materials	2021	Overview of chitosan and its modification materials for dye adsorption from 2009 to 2020	Dye	[75]
13	Chitosan-modified magnetic biochar	2021	Analyses of various modified biochars, mechanisms, dynamics, and factors influencing the adsorption process	Heavy metals	[76]
14	Chitin/chitosan, seaweeds, and seaweed-based polysaccharides	2021	Analyzing various types of marine-derived materials in water purification	Various contaminants	[77]
15	Chitosan composites	2022	A comprehensive overview of antibiotic removal, adsorption mechanisms, and influencing factors	Antibiotic residues	[78]
16	Chitosan-modified biochar	2022	Types, characterization, adsorption models, mechanisms, and applications of chitosan-biochar composites in wastewater treatment	Drug residues, dyes, phosphates, radionuclides, and perfluorochemicals,...	[79]
17	Chitosan	2022	Recent advances in the modification of chitosan-based materials by physical, chemical, and biological methods in many industries	Various contaminants	[80]
18	Shellfish waste	2022	The biochar from shellfish waste with higher adsorption capacities compared to lignocellulose biochar effectively removes emerging contaminants from aquaculture wastewater	Antibiotics, heavy metals, and excessive nutrients	[81]

## Data Availability

The authors confirm that the findings of this study are available within the article.
